# MedError: A Machine-Assisted Framework for Systematic Error Analysis in Clinical Concept Extraction

**DOI:** 10.21203/rs.3.rs-7151650/v1

**Published:** 2025-09-17

**Authors:** Hongfang Liu, Sunyang Fu, Qiuhao Lu, Jaerong Ahn, Fang Chen, Hanyun Yin, Julia Wen, Zhiyi Yue, Taylor Harrison, Jiang Jun, Xiaoyang Ruan, Ming Huang, Andrew Wen, Liwei Wang, Min Ji Kwak, Nahid Rianon, Yanshan Wang, Ruihong Huang

**Affiliations:** University of Texas Health Science Center at Houston; UTHealth Houston; UTHealth Houston; University of Texas Health Science Center at Houston; University of Texas Health Science Center at Houston; Texas A&M University; UTHealth Houston; UTHealth Houston; Mayo Clinic; UTHealth Houston; University of Texas Health Science Center at Houston; UTHealth Houston; University of Texas Health Science Center at Houston; University of Texas Health Science Center at Houston; University of Texas Health Science Center at Houston; UTHealth Houston; University of Pittsburgh; Texas A&M University

**Keywords:** Error Analysis, EHR, NLP

## Abstract

Error analysis is a critical step in evaluating and improving clinical concept extraction models, the most common clinical natural language processing (NLP) task. Unlike corpus annotation, which follows standardized protocols for creating gold-standard datasets, error analysis requires nuanced judgment grounded in both clinical expertise and NLP knowledge. This is especially important given the heterogeneity of clinical text, where variations in documentation style, note structure, and terminology can substantially influence model behavior. Despite its importance, there is currently no standardized, user-level framework to support systematic error analysis in clinical concept extraction task. In this study, we developed and validated MedError, a machine-assisted, human-in-the-loop framework designed to standardize and enhance error analysis for clinical concept extraction tasks. We collected and manually curated a corpus of 1,187 unique errors from a total of 4,237 notes across three different distinct hospitals. The error categories were defined using our previously validated error taxonomy and included 480 false negatives and 707 false positives across 25 error types and 48 clinical concept categories. We evaluated the performance of three proprietary and three open-source large language models (LLMs) in automatically classifying these errors into 26 and 15 predefined categories. We further developed a machine-assisted framework, MedError, which integrates best practices in error analysis, LLM-assisted classification and reasoning, and a user-friendly interface to enable more efficient, reproducible, and context-aware error analysis. The framework supports both single-site and federated multisite error analysis, facilitating the effective translation of clinical NLP systems into real-world settings.

## INTRODUCTION

Clinical concept extraction is a sub-task of natural language processing (NLP) that aims to extract predefined clinical concepts from unstructured text. The task includes concept mention detection and concept encoding.^1^ The primary source of information for clinical concept extraction is the electronic health records (EHR), a digital version of patients’ paper charts that provide a longitudinal, real-time record of an individual’s health information. Various NLP applications have been developed for different types of EHR notes, such as clinical notes, radiology reports, operative reports, and pathology reports, covering a wide range of clinical tasks and disease domains.^1-3^

Error analysis is a critical process for analyzing and documenting model classification errors, typically performed after NLP model runs^4, 5^. It plays an essential role across various phases of the model development, validation, and deployment cycle. During the training and validation phase, error analysis supports performance optimization by identifying failure patterns and informing model refinements. In the testing phase, it helps document limitations and improves model interpretability, providing actionable insights for future development. Another important application of error analysis arises during model deployment to new healthcare system environments or datasets. Internal model validity can shift substantially due to contextual changes across institutions^6^, and differences in EHR systems^7^, documentation patterns, clinical workflows, and data quality all contribute to heterogeneous model performance. In such scenarios, researchers assess model portability, which refers to a model’s ability to maintain performance "out of the box" or after minimal refinement when applied to data from different institutions or EHR systems.^8, 9^ Prior studies have reported F1-score degradation ranging from 4–72% across multiple disciplines, such as mental health, cardiology, and orthopedics, underscoring the variability in generalizability.^8, 9^ In these cases, detailed error analysis offers a mechanism to trace the sources of variability and identify the causes of performance degradation and further assists the development of new strategies to adapt, improve, or re-train the model for the new environment.

The process of conducting error analysis is typically performed manually by reviewing and categorizing model errors, followed by iterative refinement processes to optimize performance. Unlike corpus annotation, which is a standardized process for creating gold-standard datasets, error analysis requires evaluators to possess a strong understanding of both clinical domain knowledge and NLP techniques. Since various heterogeneous factors (e.g., documentation style, note structure, or terminology use) can significantly influence the form and format of clinical text, reaching a shared consensus is critical. As clinical NLP models are increasingly deployed in real-world settings, yet systematic approaches for error analysis remain limited.

In this study, we present MedError, a machine-assisted, open-source framework that standardizes and enhances error classification and reasoning in clinical concept extraction tasks. Our approach leverages the contextual understanding and reasoning capabilities of large language models (LLMs), which have shown remarkable performance in complex NLP tasks requiring syntactic, semantic, and conceptual abstraction. We hypothesize that the cognitive demands of error classification, such as contextual interpretation and domain-specific clinical knowledge, are well-suited to the few-shot learning capabilities of LLMs. To test this hypothesis, we deployed four validated clinical NLP models (two symbolic and two transformer-based) on 4,237 fully annotated clinical notes across three distinct hospital systems. Errors were manually labeled using a previously developed taxonomy.^5^

We evaluated six language models (three proprietary and three open-source) on their ability to classify clinical NLP errors across 26 and 15 predefined error classes. We then conducted corpus statistics and linguistic analyses to better understand model performance across heterogeneous settings, stratified by model types, error classes, and linguistic features. Based on our analysis, we derived an interactive framework, MedError, to guide the systematic error analysis assisted by an LLM-enabled open-source toolkit. To the best of our knowledge, this study is the first to benchmark LLMs for fine-grained clinical NLP error classification across a comprehensive set of predefined categories. We provide the first publicly available, de-identified dataset of expert-adjudicated clinical NLP errors and LLM rationales, which contributes a novel resource for future benchmarking and clinical NLP research. Finally, we release a lightweight web application and practical guidance for human-in-the-loop error analysis to facilitate adoption in real-world clinical NLP development and evaluation.

## RESULTS

### Error summary statistics

**Figure 1.**
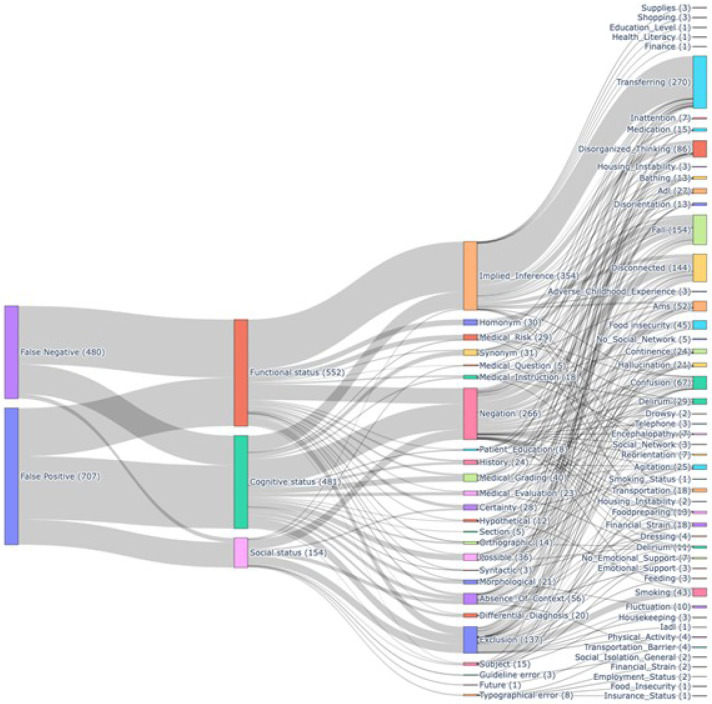
**Figure 1.** Error distribution by error class and clinical concept through Sankey diagram.

In total, we collected and manually curated 1,187 unique errors (after de-duplication) from a total of 4,237 notes, including 480 false negative and 707 false positive error types. Within this, we identified 25 error classes, with the three most frequent being implied inference, negation, and exclusion. Across the four different tasks, 48 unique clinical concept categories were identified. The interactive Sankey diagram illustrates the top error classes associated with different concept extraction sub-tasks (Fig. 1).

The distribution of error classes by false negatives and false positives variant significantly across specific tasks. For example, in identifying whether a patient needs assistance with transferring, we observed that the model produced significantly more false negatives than false positives. Most of these false negatives fell under the implied inference category, suggesting that the language used to describe transferring needs is often semantically variable and non-standardized. In contrast, the falls event sub-task revealed a broader and more diverse range of false positive error types, primarily because the word “fall” is a common homonym. This highlights the need for word sense disambiguation to correctly interpret the semantic meaning of ambiguous terms within specific clinical contexts.

A total of 552 errors were associated with the functional status task, 481 with the cognitive status task, and 154 with the social status task. Common error classes for the functional status task included implied (contextual) inference, particularly related to descriptions of transferring, falls, mobility, and other basic activities of daily living. These sub-concepts were often implied rather than explicitly stated, which suggests the need for models to possess a strong ability to infer and link contextual cues with action- oriented mentions in clinical documentation. Cognitive status tasks, which involve identifying mentions of cognitive deficits, delirium, agitation, and related conditions, exhibited a mixture of negation, inference, and semantic errors. Language describing cognitive status can range from explicit statements (e.g., “patient is delirious”) to more nuanced or implied descriptions, often paired with standardized neurocognitive assessment documentation. This variability likely contributes to the presence of typographical and morphological errors, reflecting potential challenges with spelling, syntax, and diverse expression patterns in cognition-related terms. The primary error classes for social status tasks were implied inference, negation, and exclusion. The most frequently affected concepts were food insecurity and smoking. Error types such as guideline error, absence of context, and future intent further underscore the challenges in detecting social status indicators, which are often embedded in non-clinical language, expressed implicitly, or associated with future-oriented statements.

### LLM performance comparison

We evaluated six language models on their ability to classify clinical NLP errors across 26 and 15 predefined error classes. The F1 scores for GPT-4o, O1, O3mini, LLaMA-3 8B, LLaMA-3 70B, and LLaMA-3 405B on the 26-concept task were 0.505, 0.378, 0.338, 0.118, 0.297, and 0.379, respectively. On the 15-concept task, their F1-scores were 0.637, 0.531, 0.550, 0.205, 0.352, and 0.498, respectively. From the perspective of proprietary and open-source models, GPT-4o (proprietary model) achieved the highest F1-scores for both the 26-class (0.505) and 15-class (0.637) error classification tasks. Llama-3.1-405B-Instruct-FP8, the largest open-source models, achieve an overall F1-scores of 0.379 (26-class) and 0.498 (15-class) and outperformed two proprietary models O1 and O3mini in 26-class task. The smallest LLaMA model (8B) consistently underperformed across all tasks. This overall performance results serve as the first benchmark result for comprehensive clinical NLP error classification across a diverse set of predefined error categories.

Table 1 presents the performance breakdown by three major clinical concept extraction tasks. For identifying cognitive status-related error classes, such as agitation, delirium, and disorganized thinking, where the language can be more specific and specialized compared to functional and social status tasks^10, 11^, GPT-4o yielded the strongest performance (F1 = 0.568 for 26 classes and 0.746 for 15 classes), followed by Llama405. The functional status task was dominated by the proprietary model GPT4o and O1. This task contains the highest number of implied inference error classes and rich linguistic error classes (Fig. 1), which requires the model to have both strong reasoning and computational linguistics capabilities to correctly identify errors like homonyms and orthographical errors. In the social status task, GPT-4o again outperformed others with an F1-scores of 0.487 and 0.506. Interestingly, Llama-3.1-70B-Instruct achieved the second-highest performance on the social status task, with F1-scores of 0.370 and 0.403. Overall, we observed decreased performance across models when classifying errors related to functional status and social adversity events, suggesting that these tasks likely involve more implicit, context-dependent, and semantic variable language when describing patient behaviors and social environments.

**Figure 3:**
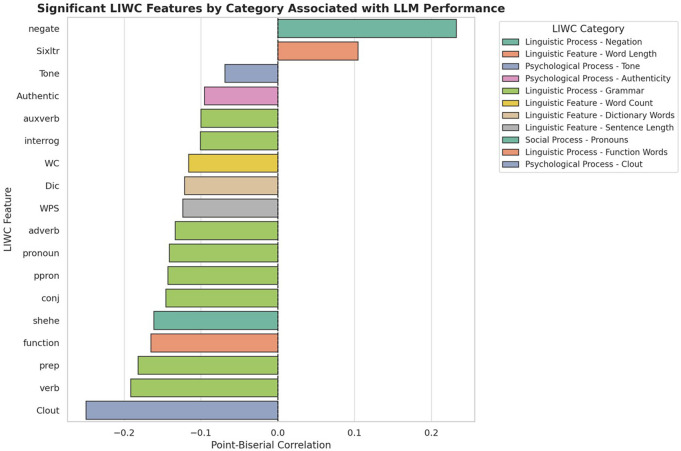
**Figure 4.** LLM performance by significantly associated linguistics features.

We further analyzed the model performance across errors created by symbolic methods versus errors generated by transformer approaches (Fig. 3a). Two donut charts present the top 10 most frequent error classes and suggest that there is high variation in the most frequent error classes between the two NLP methods. Overall, all models yielded a better performance on symbolic generated error classes compared with transformer based. This finding aligns with our expectation, as it is easier to associate the given error with its form and format based on different rule components. In contrast, for machine learning models, decisions are based on statistical inference from prior data distributions, making it more challenging to predict the causes of errors.

Figure 3b presents a comparative analysis of performance across 26 error classes for the top five performing models, GPT-4o (blue), O1 (orange), O3mini (light orange), LLaMA405 (purple) and LLaMA70 (light purple). GPT-4o consistently outperforms the other four models in a majority of categories, particularly in linguistic and contextual tasks (e.g. Orthographic, Synonym and Negation). The O1 model achieved strong performance in the tasks that require higher reasoning, including Medical_Instruction, Medical_Question, Patient_Education, and Implied_Inference. These tasks were often associated with false positive cases and marked as Exclusion. The large open-source model, LLaMA-3.1-405B-Instruct, demonstrated competitive performance in majority categories, which achieved the best performance in classifying error classes related to Morphological, Medical_Evaluation and Section. In summary, we found GPT-4o and O1 have broader generalization and contextualization strengths, and the largest open-source model can achieve competitive performance in specific subdomains. We also found a handful of error classes including Guideline, Future, Syntactic, and Typographical that yield poor agreements between model classification and human gold standard labels. Our next section will provide more in-depth analysis on these underperformed categories.

### Corpus and linguistic evaluation

Among the 91 linguistics features analyzed, we found 48 to be significantly associated with LLM performance (p < 0.05). Features that were positively associated with performance included punctuation and syntactic markers, negation, affective cues, and clinical terminology. We believe that punctuation features help LLMs by enhancing the structural clarity of sentences, possibly because punctuation improves syntactic parsing and interpretability. The WC (word count) feature showed a negative correlation, suggesting that longer sentences are more difficult for models due to the challenge of capturing long-distance dependencies. Negation terms explicitly highlight the semantic polarity or certainty of events or clinical concepts, which also correlates positively with LLM performance. Additionally, specific and explicit clinical terms (e.g., encephalopathy) tend to be semantically constrained and rely on more literal, less ambiguous language (e.g., “status come and go”), further aiding model accuracy. Conversely, several linguistic elements (e.g., clout, verb, pronoun, relative, social, female) were negatively correlated with performance. These features are likely to be associated with higher-order reasoning, implicit meaning, or context-sensitive language, which are more challenging and error-prone for current LLMs.

### Error interpretation evaluation

Leveraging an LLM in the iterative refinement processes resulted in modest but consistent improvements in model performance. Adjustments targeting false negatives led to slight increases in recall and corresponding marginal gains in F1-score. Subsequent corrections of false positives yielded incremental improvements in precision. While no single phase led to a substantial leap in metrics, the cumulative effect of targeted refinements demonstrated the potential value of focused error analysis.

### MedError: A framework to optimize machine-assisted error analysis

**Figure 6:**
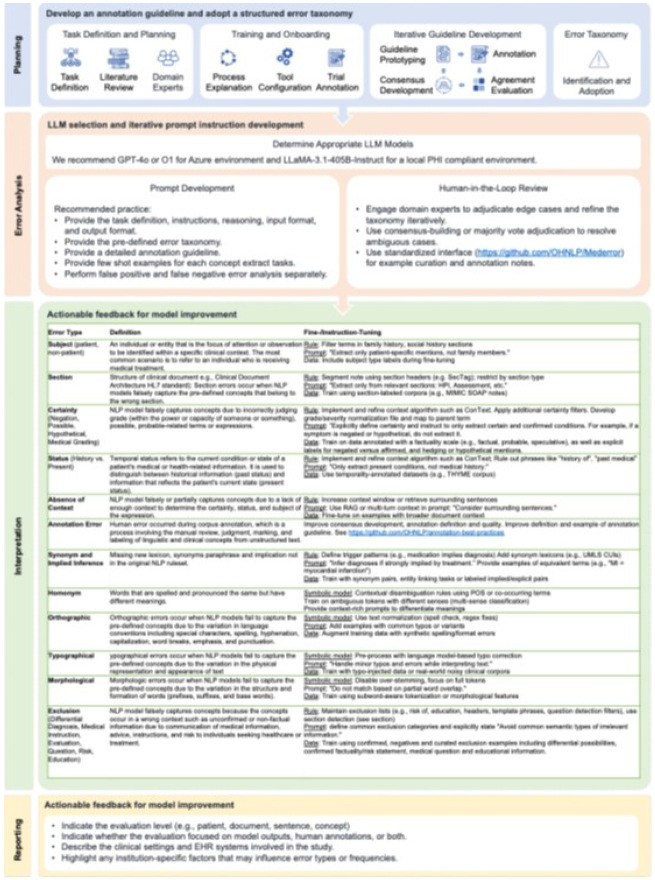
**Figure 7**. A framework for systematic human error analysis using an LLM-assisted open-source toolkit.

**Figure 7:**
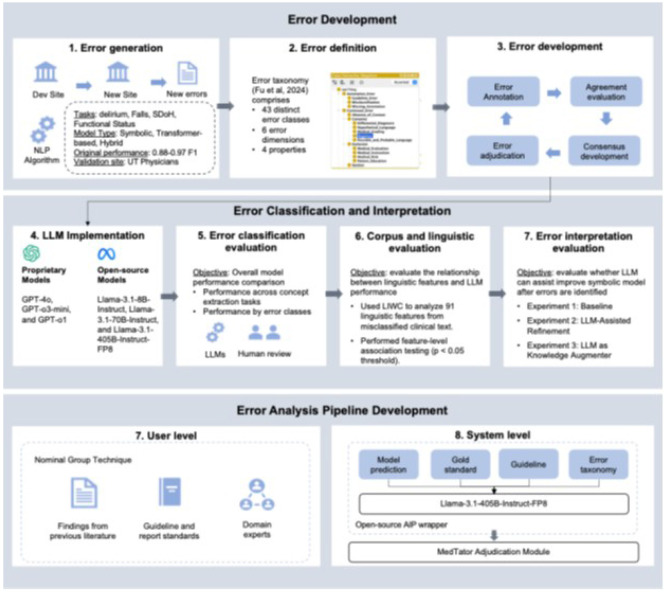
**Figure 8.** Methodology overview of the machine-assisted error analysis framework development process.

Based on our analysis, we derived an interactive framework, MedError, to guide the systematic error analysis assisted by an LLM-enabled open-source toolkit. The framework contains a web application (Fig. 6) and user process guide (Fig. 7). This web application is a lightweight, serverless frontend built using the Vite build tool and the Vue 3 JavaScript framework. Designed for efficient client-side rendering and modular component loading, it requires no backend infrastructure, once built, it can be statically hosted on any CDN or file server. The app is optimized for performance with ES module preload and code-splitting, ensuring fast loading times and minimal runtime overhead. Its architecture is fully static and scalable, making it ideal for JAMstack-style deployments on platforms like Netlify, GitHub Pages, or Vercel.

MedError integrates human-in-the-loop practices, prompt engineering, and actionable feedback to iteratively refine model performance and improve robustness in downstream applications. The input consists of model prediction outputs, gold standard annotations, annotation guidelines, and prompts encoded with our previously defined error taxonomy. Then, MedError performs automatic alignment between predicted and reference annotations, identifies discrepancies, and uses in-context learning with LLMs to assign error categories and generate reasoning chains. The results are exported in a structured format compatible with MedTator, enabling seamless integration with existing in-browser annotation workflows for expert review, adjudication, and iterative refinement. The full framework, including the codebase, prompt templates, and XML schema converters, is available at https://github.com/OHNLP/MedError.

The user process guide with recommended best practices outlined in four phases: planning, error analysis, interpretation, and reporting (Fig. 7).

- The planning phase begins with task definition, literature review, and engagement of domain experts to guide the development of annotation guidelines and the adoption of a structured error taxonomy. This phase includes training and onboarding of annotators, tool configuration, and iterative consensus-building for guideline refinement. The output is a standardized annotation protocol and error taxonomy, which will be the input for the YAML file.
- During error analysis, LLMs can be employed to assist with error identification. Recommended models include GPT-4o or OpenAI's O1 for secure cloud environments, and LLaMA-3.1-405B-Instruct for locally hosted use. Prompt development involves specifying input formats, expected reasoning chains, and task-specific instructions. Annotators are encouraged to use few-shot examples tailored to specific error types and perform false positive and false negative error analyses separately. A human-in-the-loop review process is essential for adjudicating ambiguous cases and refining the taxonomy through standardized interfaces that maintain consistency during annotation and ensure high annotation quality.
- During the error interpretation phase, errors are classified across multiple dimensions, and for each type, corresponding improvement strategies are provided. These include prompt refinements, enhancements to symbolic rules, incorporation of domain-specific fine-tuning data, and model architecture adjustments (e.g., input processing or using hybrid architecture).
- To ensure transparency and reproducibility, the findings of the error analysis should clearly specify the evaluation unit (e.g., patient, document, sentence, concept), whether the evaluation targets model predictions, human annotations, or both, the clinical settings and EHR systems involved, and any contextual or institution-specific factors that may influence error distributions.

## DISCUSSION

Our study presents a set of methods in defining, collecting, analyzing and modeling real-world errors made by four existing and distinct NLP models in clinical concept extract tasks. In total, we collected and manually curated 1,187 unique errors (after de-duplication) from three different EHR systems. The error corpus including 480 false negative and 707 false positive error types with the total of 25 error classes across 48 unique clinical concept categories. Systematic evaluation was performed on three proprietary LLMs and three open-source LLMs leveraged for automatic classification of clinical NLP errors across 26 and 15 predefined error classes. Based on our analysis, we derived a framework, MedError, to guide systematic error analysis assisted by an LLM-enabled open-source toolkit. The framework integrates human-in-the-loop practices, prompt engineering, and actionable feedback to iteratively refine model performance and improve robustness in downstream applications.

We found that task of error classification is inherently challenging due to the diverse error types, nuanced reasoning, domain knowledge, and context sensitivity required to accurately distinguish between fine grained error types. GPT-4o consistently leads across all three status (functional, cognitive and social) categories, suggesting that potential advantages such as longer context window inputs, multimodal capabilities, and the scale and coverage of training data contribute to its superior performance on nuanced classification tasks. We found that O1 shows interesting domain specific behavior by performing the best performance on Functional Status (15 class: O1 0.585 vs GPT-4o 0.578). This suggests that O1's reasoning focused architecture may be particularly suited for certain types of status assessment but struggles with the complexity of cognitive evaluation. O3mini demonstrates the most dramatic improvement when moving from 26 to 15 error classes, especially in Functional Status (0.241 to 0.580). This suggests that the model has learned meaningful representations but is overwhelmed by fine grained distinctions. The Llama series reveals clear scaling effects, with Llama405B significantly outperforms smaller variants. Llama8B's extremely poor performance (F1 scores mostly under 0.2) indicates that these tasks likely require a minimum capability threshold that very small models simply cannot reach.

Over the past two decades, numerous NLP models have been developed, yet only a small number have been successfully implemented in real-world settings or practically adopted for demonstration research.^12^ This is largely due to persistent errors, lack of transparency in model decision-making, and insufficient validation across diverse clinical contexts, all of which hinder confidence in model outputs and limit adoption. Recognizing these barriers, the NIH has recently issued several Notice of Special Interest (NOSI) to explicitly promote rigorous evaluation and transparent benchmarking of AI/NLP models in healthcare to advance real-world implementation and trustworthiness (e.g., NOT-CA-24-031). Our work directly addresses this challenge by introducing a systematic, machine-assisted framework for evaluating the clinical validity, portability, and explainability of NLP models used for clinical concept extraction. Example use cases of our framework include collaborative initiatives such as the Evolve to Next-Gen Accrual to Clinical Trials (ENACT) and the Observational Health Data Sciences and Informatics (OHDSI) networks, both of which increasingly require standardized NLP evaluation protocols and tools to ensure cross-institutional reproducibility.^13^.

Error analysis is a crucial but often under reported, and under studied area due to the majority of NLP publications being largely based on measurement study design (evaluate the validity of the model) compared with demonstration design (apply the model to derive insights). Our effort aims to address current methodological and standardization gaps by proposing an informatics framework that supports efficient, user-friendly, and standardized error analysis. This framework is designed to facilitate more rigorous evaluation of NLP applications across diverse research settings and multi-institutional EHR environments. By improving the consistency and transparency of error analysis, our approach can enhance understanding of how to effectively implement and integrate NLP tools, ultimately supporting broader adoption and meaningful use of these technologies in clinical practice.

NLP models for EHR-based concept extraction require thorough error analysis and refinement to ensure strong performance across data from different institutions. In this study, we evaluated the feasibility of leveraging LLMs to accelerate systematic error analysis. Our results indicate that while LLMs demonstrate potential feasibility in this process, their performance varies across tasks, and automated error analysis remains challenging. This study has several limitations. First, our current work focuses exclusively on clinical concept extraction tasks; other important NLP tasks such as relation extraction and coreference resolution were not addressed. Second, the deployment of LLMs in real-world settings requires PHI-compliant environments, which imposes additional technical and ethical constraints. Future work should focus on developing human-AI collaboration frameworks and innovative methodologies to enhance the effectiveness and scalability of error analysis.

## METHODS

The development of the machine-assisted error analysis framework involves a set of iterative processes including three major phases 1) error development, 2) error classification, and 3) interactive error analysis pipeline development (Figure 8). The following sections describe each step in details.

### Error generation

Despite the importance, large-scale, diverse error datasets from real-world EHRs remain scarce. Unlike public NLP datasets, EHR-derived corpora contain complex and institution-specific variations that LLMs have not been pre-exposed to, and when appropriately de-identified, they offer unique value for benchmarking LLM-assisted error analysis in novel clinical contexts. Due to potential variations in the EHR systems, documentation practices, and patient populations, the original NLP models may experience performance degradation when directly applied to a new environment. To collect real-world NLP errors, we deployed and ran four previously validated and published NLP models originally developed at Mayo Clinic and the University of Kentucky (UK) on the UT Physicians (UTP) EHR systems. Four different clinical concept extraction tasks were considered, including delirium, falls, Social Determinants of Health (SDoH), and functional status.

### Error definition

In our previous work, we developed a comprehensive error taxonomy that comprised of 43 distinct error classes, organized into 6 error dimensions and 4 properties.^5^ We conducted a thorough review of each error class and made the following modifications to the original definitions: (1) Removed "annotation error", as the focus of this study is solely on machine-generated errors rather than human annotation errors; (2) Removed "logic error", since this category requires document- or patient-level reasoning, whereas our study is limited to sentence- or paragraph-level evaluation; and (3) Removed rare error classes, including incomplete extraction, dictionary error, and normalization error, as these require access to and comparison with external knowledge bases or dictionaries for proper assessment. We release the formal definition through Github: https://github.com/OHNLP/ErrorAnalysis/blob/main/Taxonomy/error_taxonomy.md

### Error development

The error development workflow was guided by the TRUST process ^6, 18^, which has been adopted by the National Center for Data to Health (CD2H) as best practices. Briefly, the process involves a set of quality control iteration (e.g., annotation training, double annotation, IAA measurement) consensus development, and adjudication. All cases studies followed a standardized annotation guideline ^10, 16, 19, 20^. We applied our previously established error definition to these newly generated errors through a formal annotation process performed by a team of trained abstractors (S.F., J.A., F.C., and Z.Y). The initial phase and second phase agreement ratios are 0.60 and 0.68, respectively. Quality check for all error cases was conducted by S.F. with 10+ years of experience in gold standard development and NLP methodology. Challenging cases and error annotation questions were described in Supplementary Appendix.

### LLM implementation

We implemented GPT-4o, GPT-o3-mini, and GPT-o1 under the PHI-compliant Azure environment and Llama-3.1-8B-Instruct, Llama-3.1-70B-Instruct, and Llama-3.1-405B-Instruct-FP8 architectures within a local PHI environment. We employed in-context learning by providing error taxonomy, a task-specific guideline, examples, and structured prompts to guide model responses without requiring fine-tuning.

### LLM evaluation

Model performance was evaluated against human annotations using standard metrics, including precision, recall, and F1-score. To gain deeper insights into model behavior, we conducted a detailed error analysis by qualitatively reviewing both the misclassification outputs and the explanatory reasoning generated by large language models (LLMs). Our evaluation included: (1) overall performance comparison across models, (2) task-specific performance for different clinical concept extraction scenarios, and (3) error-specific performance stratified by predefined error classes.

### Corpus and linguistic evaluation

LIWC Model. We employed the LIWC (Linguistic Inquiry and Word Count) text analysis tool to extract well-validated psycholinguistic lexicons. LIWC leveraged a dictionary lookup approach to match the words that make up each text entry to sentiment-specific dictionaries. The tool was originally developed for psychology research. Due to its high effectiveness in emotion extraction and sentiment analysis, the tool has been widely adopted for various domains such as social computing and biomedical informatics. Based on previously established definitions, we examined a total of 91 linguistic and semantic features.

### Error interpretation evaluation

Previous tasks have focused on evaluating LLMs for identifying or predicting different error classes. In this task, we aim to assess how LLMs can support the refinement and optimization of model performance using the previous identified errors. In order to make the scope manageable, we only focus on a symbolic model and two extraction tasks. For the symbolic framework, we adopted a rule-based framework, MedTagger, an open-source information extraction pipeline based on the UIMA (Unstructured Information Management Architecture) framework, to facilitate clinical text processing and evaluation of LLM generated lexicons against a gold standard. The study involved three experiments using OpenAI ChatGPT 4o:

- Phase 1 (Baseline): Use annotated lexicons and phrases only from the training data.
- Phase 2 - LLM as a knowledge augmenter: The third experiment fully automated regex generation, with the LLM creating the regex list based on the task definition and a small set of training examples (3.1). The second phase of this experiment focused on capturing linguistic variations, including orthographic, semantic, synonyms, implied inferences, and typographical differences (3.2).
- Phase 3 - LLM-assisted error refinement: The LLM suggested and refined regex based on the identified false positives (2.1) and false negatives (2.2)

The results from each experiment were then compared to the gold standard to assess MedTagger’s performance across different regex-generation methods. Overlapping index ranges were classified as true positives, while non-overlapping ones were considered false positives or false negatives.

### Error analysis pipeline development

Based on the findings of the study, we proposed a user guide and open-source pipeline to enhance the usability, efficiency, and standardization of the error analysis process. To support scalable and interoperable error analysis in clinical NLP, we have developed and released an open-source framework that automates error detection, classification, and explanation using large language models.

## Supplementary Material

This is a list of supplementary files associated with this preprint. Click to download.

Appendix1Prompt.docx

Appendix2ErrorGuideline.docx

## Figures and Tables

**Figure 2:**
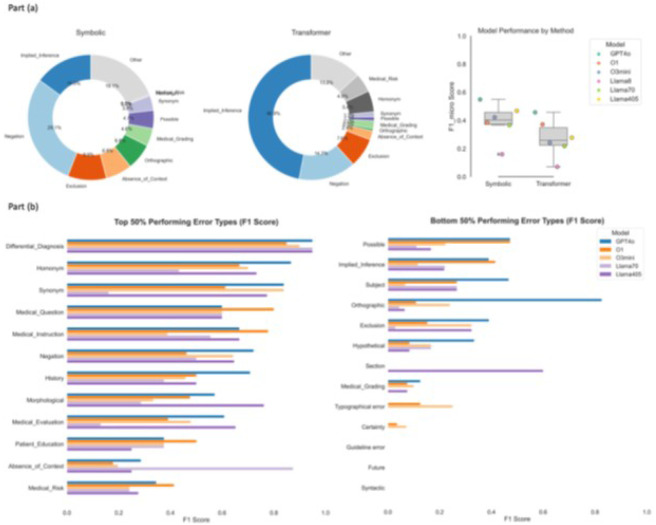
**Figure 3.** Summary of the top three performing models, GPT-4o (blue), O1 (orange), O3mini (light orange), LLaMA405 (purple) and LLaMA70 (light purple), by error classes. See appendix for detailed definition for all error classes.

**Figure 4:**
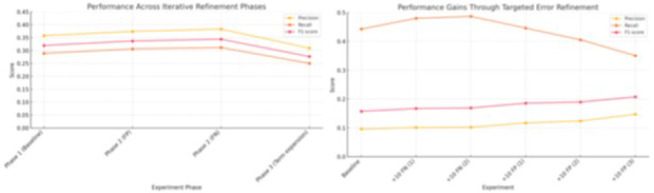
**Figure 5.** Overview of model performance change after LLM-assisted error refinement

**Figure 5:**
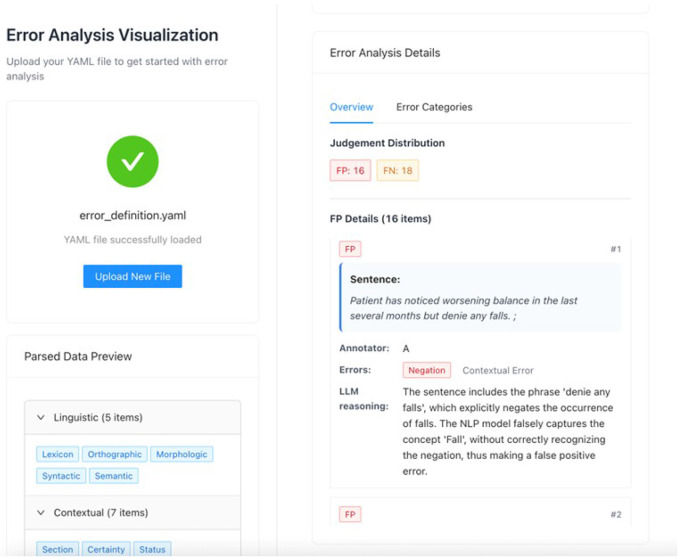
**Figure 6**. User interface web application of MedError.

**Table 1:** Overall performance comparison across six models.

Task	Model	F1-score (26 error classes)	F1-score (15 error classes)
Cognitive Status (n = 481)	GPT4o	**0.568**	**0.746**
	O1	0.412	0.530
	O3mini	0.443	0.576
	Llama8	0.183	0.304
	Llama70	0.366	0.437
	Llama405	0.511	0.684
Functional Status (n = 552)	GPT4o	**0.457**	0.578
	O1	0.371	**0.585**
	O3mini	0.241	0.580
	Llama8	0.071	0.145
	Llama70	0.217	0.264
	Llama405	0.277	0.380
Social Status (n = 154)	GPT4o	**0.487**	**0.506**
	O1	0.299	0.338
	O3mini	0.357	0.364
	Llama8	0.084	0.110
	Llama70	0.370	0.403
	Llama405	0.331	0.338

**Table 1.** Summary of concept extraction tasks, environment settings, and model types for error collection

Task Name	Original Site	Applied Site	# of Notes	Model Type	Reference
Cognitive Status	Mayo Clinic	UT Physicians	654	Symbolic	^14^
		Memorial Hermann Health System	192		
Functional Status	Mayo Clinic	UT Physicians	2047	Transformer, Hybrid	^15, 16^
		Memorial Hermann Health System	944		
Social Status	University of Kentucky	UT Physicians	200	Symbolic, Transformer	^17^
		Texas children's hospital	200		

## Data Availability

All data generated or analyzed during this study are included in this published article and its supplementary files. Data access: Interested parties can request access by contacting Center for TEAM-AI and are required to sign and remain compliant with a Data Use Agreement under the UTP Center (natural language processing) Program.
